# Autoinhibition and activation mechanisms of the eukaryotic lipid flippase Drs2p-Cdc50p

**DOI:** 10.1038/s41467-019-12191-9

**Published:** 2019-09-12

**Authors:** Lin Bai, Amanda Kovach, Qinglong You, Hao-Chi Hsu, Gongpu Zhao, Huilin Li

**Affiliations:** 1 Structural Biology Program, Van Andel Research Institute, Grand Rapids, MI 49503 USA; 2 David Van Andel Advanced Cryo-Electron Microscopy Suite, Van Andel Research Institute, Grand Rapids, MI 49503 USA

**Keywords:** Membrane proteins, Cryoelectron microscopy, Biophysics

## Abstract

The heterodimeric eukaryotic Drs2p-Cdc50p complex is a lipid flippase that maintains cell membrane asymmetry. The enzyme complex exists in an autoinhibited form in the absence of an activator and is specifically activated by phosphatidylinositol-4-phosphate (PI4P), although the underlying mechanisms have been unclear. Here we report the cryo-EM structures of intact Drs2p-Cdc50p isolated from *S*. *cerevisiae* in apo form and in the PI4P-activated form at 2.8 Å and 3.3 Å resolution, respectively. The structures reveal that the Drs2p C-terminus lines a long groove in the cytosolic regulatory region to inhibit the flippase activity. PIP4 binding in a cytosol-proximal membrane region triggers a 90° rotation of a cytosolic helix switch that is located just upstream of the inhibitory C-terminal peptide. The rotation of the helix switch dislodges the C-terminus from the regulatory region, activating the flippase.

## Abstract (web summary)

The heterodimeric eukaryotic Drs2p-Cdc50p complex is a lipid flippase that maintains cell membrane asymmetry and its autoinhibition is released by PI4P binding. Here authors show cryo-EM structures of Drs2p-Cdc50p in apo and PI4P-activated form which reveal the structural changes upon PI4P binding.

## Introduction

The yeast lipid flippase Drs2p-Cdc50p is primarily found in the trans-Golgi network and is involved in protein trafficking between that network, the plasma membrane, and endosomes^1^. Drs2p belongs to a large family of Type IV P-type ATPases (P4 ATPases)^2^. These enzymes translocate specific phospholipids from the extracellular side of the plasma membrane or from the lumenal side of internal organelles to the cytosolic side, creating and maintaining lipid compositional asymmetry in eukaryotic cell membranes. Drs2p has an architecture similar to that of several cation-transporting P-type ATPases, having an actuator (A) domain, a nucleotide-binding (N) domain, a phosphorylation (P) domain, and a transmembrane domain (TMD) of 10 transmembrane helixes (TMs)^3^. The N-terminal and C-terminal peptides of Drs2p inhibit the enzyme activity^4,5^. Cdc50p functions as a chaperone; its association with Drs2p is required for stability and ER export^1,6,7^, but Cdc50p may also contribute to Drs2p activity via an unknown mechanism^8^.

Drs2p-Cdc50p alone is in an autoinhibited inactive form^4,9^. Drs2p-Cdc50p is activated by the binding of phosphatidylinositol 4-phosphate (PI4P), the Arf guanine nucleotide exchange factor Gea2p, and the Arf-related small-GTP-binding protein Arl1p to the C-terminal region of Drs2p^9,10^. Phosphatidylserine appears to be the primary substrate of Drs2p-Cdc50p^11–13^, although phosphatidylethanolamine can also be transported to some extent^14^. We sought to understand how the enzyme complex inhibits itself and how PI4P activates the flippase activity by determining the cryo-EM structures of the Drs2p-Cdc50p complex in the autoinhibited apo form and in the PI4P-activated form. Because we use the intact complex rather than a C-terminal truncated form, the conformational changes we observe in Drs2p upon PI4P incubation may be a more faithful representation of the activation events that occur in vivo.

## Results

### Cryo-EM of autoinhibited and PI4P-activated Drs2p-Cdc50p

**Fig. 1:**
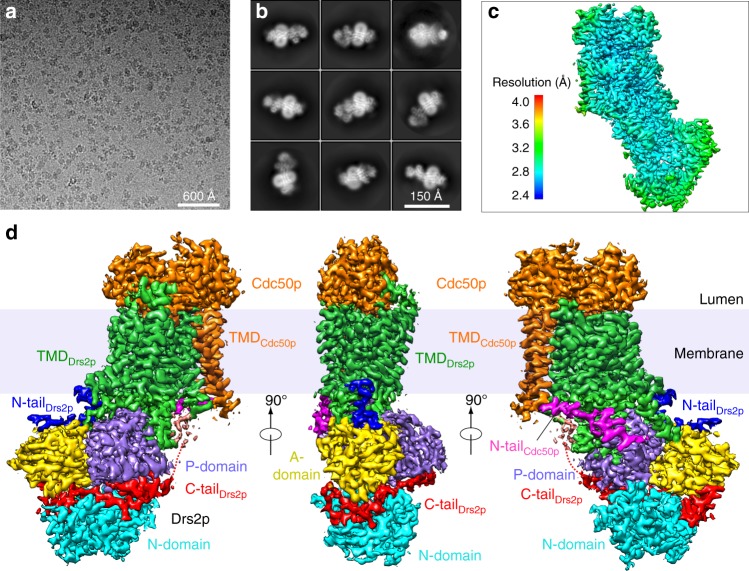
Cryo-EM of the apo Drs2p-Cdc50p complex. **a** Representative electron micrograph. **b** Selected reference-free 2D class averages. **c** Local resolution map. **d** Surface rendering of the 3D map colored by protein subunits and major domains and N- or C-terminal peptides

To prepare the endogenous Drs2p-Cdc50p complex, we added a triple FLAG tag to the C-terminus of the *S*. *cerevisiae* Drs2p and purified the complex from baker’s yeast using an affinity column followed by size-exclusion chromatography (Supplementary Table 1). The presence of both Drs2p and Cdc50p was confirmed by SDS-PAGE and mass spectrometry (Supplementary Fig. 1). We first performed single-particle cryo-EM directly on the purified Drs2p-Cdc50p. We collected a cryo-EM data set of 2970 micrographs in a Titan Krios microscope, resulting in about 1,000,000 raw particles. After 2D and 3D classifications, some 640,000 particles were selected for 3D reconstruction and refinement, leading to a 3D map in the apo form at 2.8 Å resolution (Fig. 1, Supplementary Fig. 2). The high quality of the 3D map enabled ab initio modeling of Cdc50p, which lacked a homolog structure. Model building of Drs2p was aided by available P2 ATPase structures, such as the gastric proton-pumping ATPase^15^. The model was refined to good statistics (Supplementary Table 2), and it fit well with the density map (Supplementary Fig. 3). We found the purified Drs2p-Cdc50p was free of any ligand in an autoinhibited apo state. To understand its activation mechanism, we incubated the Drs2p-Cdc50p with PI4P overnight and then performed single-particle cryo-EM on the enzyme complex. We derived a 3.3-Å resolution 3D map of the complex in an activated conformation from 498,745 particles that were selected from 4717 raw electron micrographs (Supplementary Fig. 4). The corresponding model was built based on the apo Drs2p-Cdc50p structure. We found that PI4P diffuses away from its binding pocket in the transmembrane region upon activating the Drs2p ATPase. We went on to generate a series of point mutations around the putative PI4P binding site and demonstrated that these modifications indeed affect the enzyme functions, as described in the following sections.

### Overall structure

**Fig. 2:**
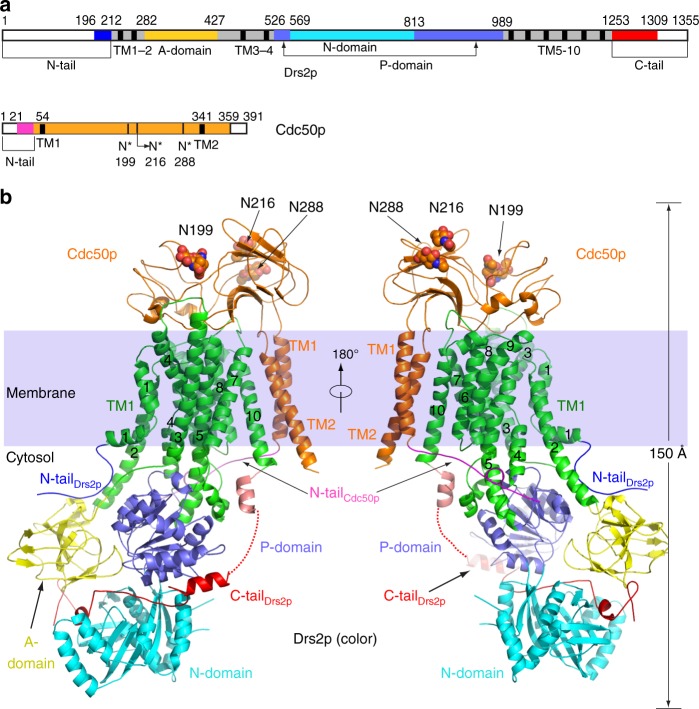
Molecular architecture of the *Saccharomyces cerevisiae* Drs2p-Cdc50p complex. **a** Domain structures of the full-length Drs2p and Cdc50p. Regions not observed are in white. **b** A frontside and a backside view of the apo Drs2p-Cdc50p structure viewed from the membrane plane. The transmembrane domain of Drs2p is shown in green. The A, N, and P domains of Drs2p are shown in yellow, cyan, and purple, respectively. The autoinhibitory C-tail of Drs2p is shown in red. The regulatory N-tails of Drs2p and Cdc50p are shown in blue and pink, respectively. Cdc50p is shown in orange. N-glycans are shown as spheres

The yeast Drs2p is a large protein with 1355 residues (Fig. 2a). As expected, Drs2p contains 10 transmembrane helices, with the cytosolic A domain being inserted between TM2 and TM3, and the cytosolic P and N domains inserted between TM4 and TM5. In fact, the N domain is inserted within the larger P domain, splitting the P domain into two parts in sequence (Fig. 2a, b). Preceding the TM1 is a nearly 200-residue-long N-terminal peptide that is largely disordered and invisible in our cryo-EM maps, except for a 17-residue proximal N-terminal peptide (Phe-196 to Thr-212) that is stabilized by binding to the cytosolic regulatory region of Drs2p. Following the last transmembrane helix, TM10, is another 125-residue-long C-terminal peptide (Glu-1231 to Ile-1355). The first 60% of this peptide (Glu1231 to Ala-1309) is largely ordered in the apo structure by its extensive interactions with the cytosolic regulatory region of Drs2p (described below). Indeed, this region is responsible for inhibition, as its removal relives the autoinhibition of Drs2p^5^. The last 46 residues (Asn-1310 to Ile-1355) are disordered in the cryo-EM map. The disordered extreme C-terminal region may not to be functionally important and does not participate in the autoinhibition of the enzyme, because removal of this peptide by truncation does not stimulate the Drs2p activity. In other words, the truncated Drs2p remains in the inhibited state as long as the first 60% of the ordered C-terminal peptide is present^5^.

The Drs2p-Cdc50p complex has a highly elongated architecture that is about 150 Å tall and 65 Å wide (Fig. 2a, b, Supplementary Movie 1). The Drs2p subunit is a three-tiered structure, with a large transmembrane domain as the first (top) tier, followed by the A and the P domains as the second (middle) tier, and the N domain at the bottom as the third tier. Cdc50p primarily binds to the top of the TMD of Drs2p from the lumenal side, making the entire Drs2p-Cdc50p complex a four-tiered structure, which accounts for its height. Drs2p shares a 22% sequence identity with the sarco(endo)plasmic reticulum Ca^2+^-ATPase (SERCA), which is a P2 ATPase whose structure has been extensively characterized in the literature^16^. We compared the apo Drs2p structure with those of the SERCA ATPase (Supplementary Figs. 5 and 6), and found that our Drs2p structure differed from all four major conformations (E1, E1-P, E2, and E2-P) of SERCA^16^ (Supplementary Fig. 5a, d). The TMDs and P domain of Drs2p were in a similar position and orientation as in SERCA ATPase in the E2 or E2-P state. However, this does not mean that the apo Drs2p is in an E2 or E2-P state, because its A and N domains are differently positioned. We suggest the apo Drs2p is in a unique autoinhibited conformation that is distinct from any of the four active states of the SERCA ATPase.

Cdc50p has one transmembrane helix at the N-terminal region and another at the C-terminal region. These two TMs come together and pack against TM10 of Drs2p (Fig. 2b). Preceding TM1 of Cdc50p is a 40-residue N-terminal peptide. The first half of this peptide is disordered, but the second half is stabilized by binding the P domain and the cytosol-proximal transmembrane region of Drs2p. Following the last TM helix, TM2, is a disordered 31-residue C-terminal peptide. In between the two TMs of Cdc50p is a lumenal domain with two subdomains. The first subdomain is located right above TM1-2 of the protein and is composed of two 5-stranded β-sheets stacked against each other. The second subdomain is largely composed of short α-helices and loops and is situated above the TMD of Drs2p. The lumenal domain of Cdc50p contains two disulfide bonds (Cys-80 with Cys-123 and Cys-176 with Cys-190), one in each of the two subdomains (Supplementary Fig. 7). They likely stabilize the structure in the lumen of the trans-Golgi network. Cdc50p contains two additional Cys residues in the middle of the transmembrane region, one in TM1 (Cys-54) and the other in TM2 (Cys-343). These two cysteines do not form a disulfide bond, because they face away from each other, with Cys-54 pointing outward to the lipid environment and Cys-343 facing the TM10 of Drs2p. Cdc50p contains five NxS/T N-glycosylation motifs, among which three were found to be actually modified, at Asn-199, Asn-216, and Asn-288 (Fig. 2b, Supplementary Fig. 3). These glycans, along with the two disulfide bonds, stabilize the Cdc50p structure in the lumen.

### Autoinhibitory and regulatory mechanism of Drs2p-Cdc50p

**Fig. 3:**
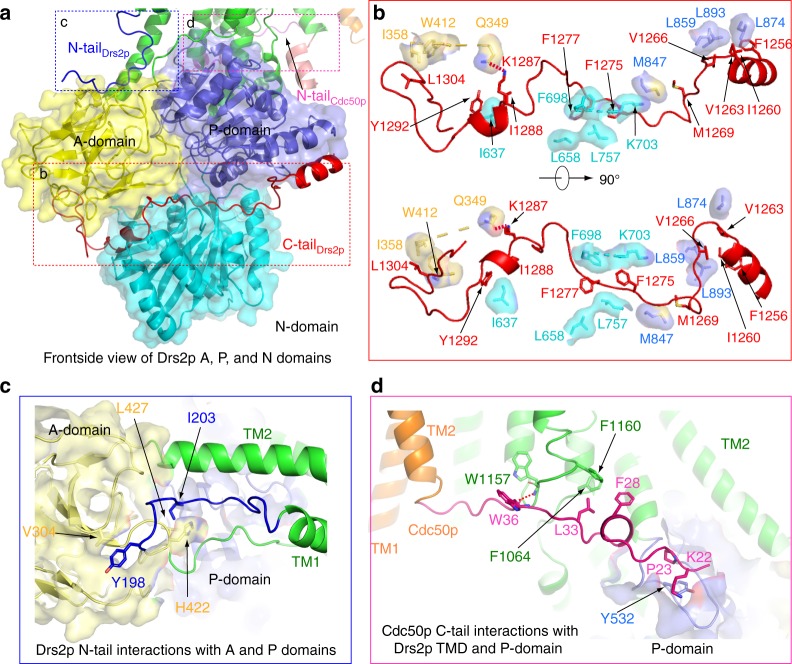
Regulatory interactions in Drs2p-Cdc50p. **a** Overview of the A, N, and P domains of Drs2p showing the Drs2p C-tail (red cartoon), N-tail (blue cartoon), and the Cdc50p N-tail (pink cartoon). **b** Detailed interactions of the Drs2p C-tail with the A, N, and P domains of Drs2p. **c** Detailed interactions between the Drs2p N-tail with the A and P domains of Drs2p. **d** Interactions between Cdc50p N-tail and the TMD and the P domain of Drs2p

Previous studies showed that the apo Drs2p is in a self-inhibited state, mainly by its C-terminal peptides^4,5^. In our apo Drs2p structure, the ordered region of the Drs2p C-tail is 57 residues long and fills an extended horizontal groove between the second-tier A and P domains and the third-tier N domain. Hence, the Drs2p C-tail makes extensive contact with all three cytosolic regulatory domains (Fig. 3a, b). The C-tail interaction with the P domain is hydrophobic, mediated by Phe-1256, Ile-1260, Val-1263, Val-1266, and Met-1269 of the C-tail and Met-847, Leu-859, Leu-874, and Leu-893 of the P domain. The C-tail interaction with the N domain is also hydrophobic, involving Phe-1275, Phe-1277, Ile-1288, and Tyr-1292 of the C-tail and Ile-637, Leu-658, Leu-757, and Lys-703 of the N domain. Note that Lys-703 of the N domain forms a cation-π interaction with the C-tail Phe-1275. The interaction between the C-tail and the A domain has a mixed nature: Lys-1287 of the C-tail forms a hydrogen bond with the A domain’s Gln-349, while the C-tail Leu-1304 hydrophobically interacts with Ile-358 and Trp-412 of the A domain.

**Fig. 4:**
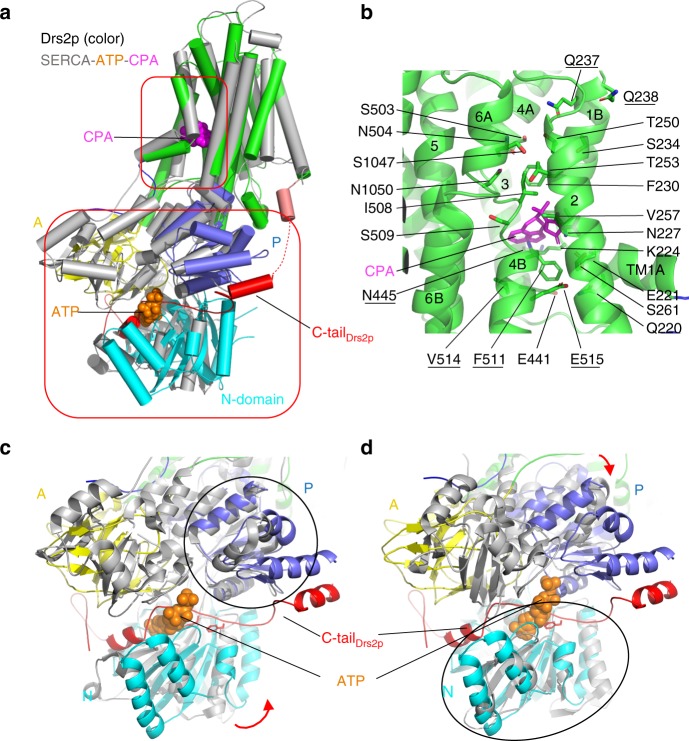
A putative substrate lipid-binding site and the ATP-binding site in Drs2p. **a** Structural comparison between Drs2p (color) and the SERCA ATPase (PDB 3FPB; gray) in complex with cyclopiazonic acid (CPA) and ATP (which stabilizes the ATPase in the E2-P state), by aligning their respective TMDs. Regions in the top box highlight the substrate lipid-binding site and in the lower box highlight the ATP-binding site in the SERCA ATPase. **b** A putative substrate lipid-binding site in Drs2p, with residues lining the pocket shown as sticks. Previous mutagenesis showed that the underscored residues determine the specificity for phosphatidylserine transport in Drs2p^14^. **c**–**d** Structural superposition of Drs2p and the SERCA ATPase by aligning their respective P domains (**c**) or N domains (**d**). The bound ATP in the SERCA ATPase is shown as spheres. The corresponding nucleotide-binding site in Drs2p is occupied by the C-terminal peptide of Drs2p. The curved red arrows in panels (**c**) and (**d**) show the domain rotations between the two structures

The catalytic cycle of a P-type ATPase requires ATP binding in the nucleotide-binding pocket in the N domain, followed by phosphorylation of an Asp in the P domain, leading to a rotation of the A domain that subsequently modulates the conformation of TMD and thus the cation translocation activity^17^. A structural alignment with the SERCA ATPase in complex with ATP showed that the nucleotide-binding site was occluded by Phe-1275 and Phe-1277 in the C-tail (Fig. 4a, c, d). The C-tail appears to cement the A, N, and P domains, preventing ATP binding by inserting these two phenylalanine residues into the nucleotide pocket and physically separating the N domain from the P domain (Fig. 3b), so that Asp-560 of the P domain cannot be phosphorylated. This structural feature readily explains why Drs2p in the apo form is fully inhibited by its C-tail^4,5^. Many plasma membrane calcium ATPases (PMCA) are also regulated by their C-terminal peptides^18^. In fact, the structure of the human PMCA1 in complex with its obligatory subunit neuroplastin was recently reported, but the inhibitory C-terminus was not visualized in the 4.1-Å resolution cryo-EM 3D map^19^.

The N-terminal peptide of Drs2p is also known to be inhibitory, although its effect is much less severe than that of the C-tail^4,5^. In our structure, most of the N-terminal peptide is disordered except for the 17-residue region (Phe-196 to Thr-212) before reaching TM1. The ordered N-terminal peptide extends over both the P and A domains (Fig. 3c). In particular, this N-peptide engages in two hydrophobic interactions with the A domain, one involving Ile-203 of the N-peptide and Leu-427 and His-422 of A domain, and the other involving Tyr-198 of the N-peptide and Val-304 of A domain. These interactions likely account for the observed inhibitory effect of the N-terminus on Drs2p activity.

The binding of the cytosolic N-terminal peptide of Cdc50p to both the TMD and the P domain of Drs2p was unexpected (Fig. 4d). The interactions between the Cdc50p N-terminal peptide and the Drs2p TMD involve a hydrogen bond between the main-chain nitrogen of Cdc50p Trp-38 and the main-chain carbonyl oxygen of Drs2p Trp-1157, as well as a hydrophobic contact between Cdc50p Leu-33 and Drs2p Phe-1160. The Cdc50p N-terminal peptide reaches further and makes another hydrophobic interaction with the P domain of Drs2p that involves Cdc50p Pro-23 and Lys-22, and the Tyr-532 of the Drs2p P domain. The Cdc50p Lys-22 makes a cation-π interaction with Tyr-532. Cdc50p is known to be required for the exit of Drs2p from the ER^1^; whether the observed interactions suggest a regulatory role for Cdc50p in the catalytic cycle of Drs2p requires further investigation.

### A putative substrate binding site

Although Drs2p and the SERCA ATPase transport very different substrates, the high similarity of the TMDs may suggest a similar substrate-transporting path, which is composed of TM1, TM2, TM3, TM4, and TM6 in the SERCA ATPase (Fig. 4a, b). The substrate-transporting path is conserved in many metal-translocating P type ATPases for which the structures are known^17,20,21^. Notably, both Drs2p and SERCA ATPase share a similarity in terms of a broken or kinked TM1, TM4, and TM6. We superimposed the structures of Drs2p on the SERCA ATPase stabilized in a E2-P like state by cyclopiazonic acid (CPA)^22^ to identify a putative substrate-transporting in Drs2p (Fig. 4a, b). This path in Drs2p features hydrophobic residues (Phe-230, Val-257, Ile-508, and Phe-511) in the middle and polar residues in both ends (Gln-237, Gln238, Thr-250, Ser503, Asn-504, Ser-1047, and Asn-1050 in the lumenal side; Gln-220, Glu-221, Lys-224, Asn-227, Ser-261, Glu-441, Asn-445, and Glu-515 in the cytosolic side) (Fig. 4b). The polar residues are separated into two clusters, which may represent two binding sites for the phosphatidylserine head group, one near cytoplasmic side and the other close to lumenal side of the TMDs.

Consistent with the structural features, residues in this putative substrate-transporting path (including Gln-237, Gln-238, Asn-445, Phe-511, Val-514, and Glu-515) have been demonstrated to determine the specificity for phosphatidylserine transport^14^. Our observation agrees with the proposed two gates mechanism, in which two sites sequentially bind and select the lipid headgroup on opposite sides of the membrane. Furthermore, TM2 precedes and is subjected to the actuating action of A domain, and TM4 immediately precedes N and P domains and is subjected to regulation by nucleotide binding in the N domain and phosphorylation in the P domain. In our apo Drs2p structure, the putative substrate path is too narrow to accommodate a phosphatidylserine, but the path may widen upon nucleotide and substrate binding^20^.

### The activated conformation

**Fig. 5:**
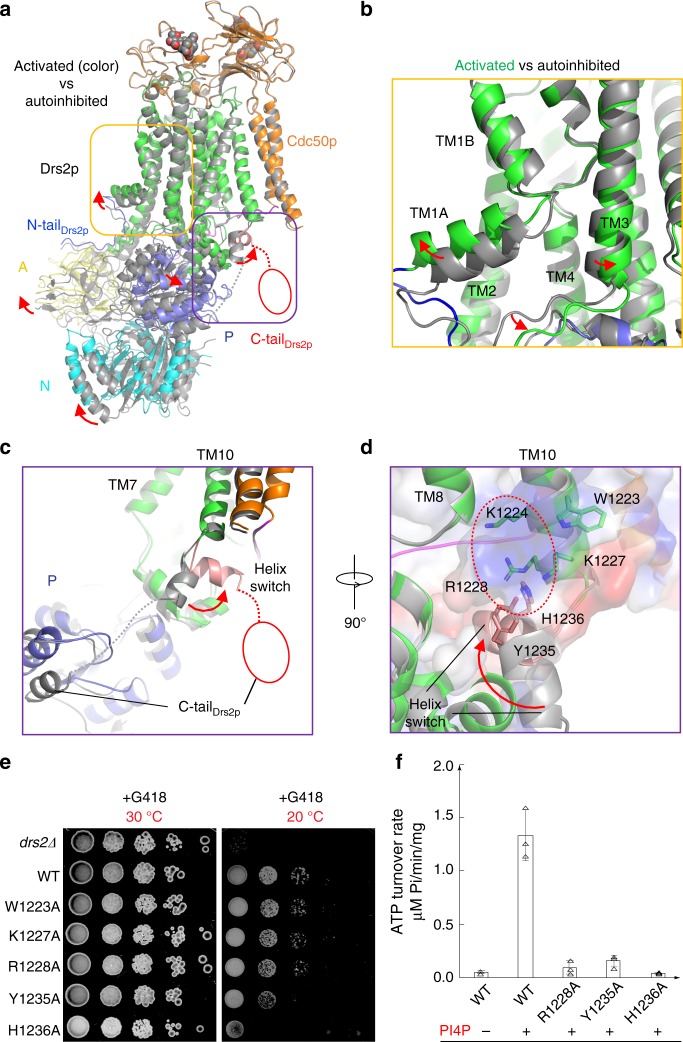
The activated conformation of Drs2p-Cdc50p. **a** Structural comparison of Drs2p-Cdc50p in the apo and active conformations. The yellow and purple boxes highlight the substrate-transporting path and the C-terminal helix switch in Drs2p. **b** A putative substrate-transporting path in Drs2p in the apo and active conformations. **c**–**d** The putative PI4P binding site of Drs2p in the apo and active conformations. The helix switch of Drs2p rotates by about 90° toward the right in going from the apo to the active conformation. **e** Complementation of *drs2*Δ cells (selected by G418) with plasmids carrying either wild-type DRS2 (WT), *drs2*-W1223A, *drs2*-K1227A, *drs2*-R1228A, *drs2*-Y1235A, *drs2*-H1236A, or an empty plasmid (*drs2*Δ). Cells were serially diluted and incubated at 30 and 20 °C for 2 days. **f** The ATPase activity of the wild-type Drs2p (WT) and mutant enzyme complexes preincubated with PI4P. Each triangle represents a data point. Error bars are standard deviations estimated from three independent measurements

Previous studies have shown that Drs2p-Cdc50p can be activated in vitro by PI4P^9,10^. We obtained the activated complex by incubating the purified sample with 26 μM PI4P for 15 h at 4 °C, resulting in a cryo-EM structure determined at 3.3 Å resolution (Fig. 5a–d). We found that in vitro activation by PI4P was a very slow process; incubating the protein for a shorter period, say, 1-2 h, led to a mixture of activated and inactive conformations. Compared with the autoinhibited apo form, the active Drs2p-Cdc50p shows two major conformational changes (Fig. 5a–d): (1) the inhibitory C-tail of Drs2p is fully released from the extended horizontal groove in the regulatory A, N, and P domains of Drs2p and becomes disordered, and (2) the putative substrate-translocating path (see Fig. 4b) becomes more open, especially in the cytosolic side (Fig. 5b).

As discussed above, the Drs2p C-tail inhibits the Drs2p activity by interacting extensively with the A, N, and P domains of Drs2p and occupying the ATP-binding pocket. In the structure of the active Drs2p-Cdc50p, the entire Drs2p C-tail becomes disordered, thus vacating the ATP-binding pocket of Drs2p previously occupied by Phe-1275 and Phe-1277 (Fig. 5a). The A, N, and P domains in the activated conformation have all undergone rigid-body movement relative to the inhibited apo structure. Notably, the A domain, previously stabilized by the last stretch of the ordered inhibitory C-terminal peptide, has moved about 6 Å away from the P domain and has become partially flexible, as indicated by its weakened EM density (Supplementary Fig. 4). The A domain movement exposes the nucleotide-binding pocket in the N domain at the junction of the A, N, and P domains, perhaps priming the Drs2p structure for ATP entry, which is required for the lipid-flipping activity of the enzyme complex. The N domain has rotated about 15° toward the A domain, pivoting around the contacting point between P and N domains, while the P domain has moved by about 4 Å in a direction opposite the A domain movement (Fig. 5a). Because the A and P domains are extensively connected with the first half of the transmembrane domain (TM1-6) in the substrate-transporting path, the upward movement of the A domain has moved TM1A upward by about 4 Å and the cytoplasmic end of TM2 to the left by 2–3 Å. Further, the rightward movement of the P domain has moved the cytosolic ends of TM3-6 by 1–2 Å, thereby resulting in a much more open substrate lipid-binding site in the cytosolic side (Fig. 5a, b). Therefore, the PI4P-activated Drs2p is in a configuration ready to deposit the substrate phosphatidylserine (PS) into the cytoplasmic leaflet of the bilayer.

The ordered C-terminal inhibitory peptide is connected to the last transmembrane helix, TM10, by a short α-helix (see Fig. 2b). In the PI4P-activated structure, we found that this α-helix has swung 90°, away from the cytosolic regulatory A, N, and P domains on the left. Conceivably, such a swing motion will pull the inhibitory C-terminal peptide out of its horizontal groove and release it into solvent, hence releasing the inhibitory grip of the C-tail on the regulatory region. For this reason, we have termed this short α-helix the helix switch (Fig. 5c, Supplementary Fig. 8).

### The putative PI4P binding site in Drs2p

As we mentioned above, PI4P, which activates the enzyme, is absent in the cryo-EM map, suggesting that after it completes the activation of the enzyme, PI4P diffuses away from the binding pocket. We observed a highly positively charged pocket close to and just above the helix switch, which is lined by Trp-1223, Lys-1224, Lys-1227, Arg-1228, Tyr-1235, and His-1236 (Fig. 5d). Because of (1) the positive charge of this pocket, (2) its proximity to the helix switch, and (3) knowing that the PI4P binding site in Drs2p is at this particular C-terminal region^9^, we suggest that this is the PI4P binding pocket and that PI4P binding at this site allosterically regulates the flippase activity of the enzyme complex^5,9,23^. Because the substrate PS is also negatively charged, we do not exclude the possibility that it may also bind in this pocket.

We further investigated the functional significance of this putative PI4P binding pocket by mutagenesis. We took advantage of a yeast *drs2*Δ strain which has a cold-sensitive growth-defect phenotype; this deficiency can be complemented by a plasmid carrying the wild-type *drs2* gene. We introduced five individual mutations in the putative PI4P binding site (W1223A, K1227A, R1228A, Y1235A, and H1236A) and performed complementation assays on *drs2*Δ cells. We found that only Y1235A and H1236A significantly disrupted Drs2 function in vivo, as these mutants were unable to rescue the *drs2*Δ yeast growth at 20 °C than those plasmids carrying the WT *drs2* (Fig. 5e). R1228A partially lost the ability to rescue the *drs2*Δ yeast. Furthermore, we purified the R1228A, Y1235A, and H1236A mutant Drs2p-Cdc50p complexes and measured their ATP hydrolysis activity, and found that R1228A, Y1235A, and H1236A all ablated PI4P activation of the purified Drs2p-Cdc50p complex (Fig. 5f), suggesting that PI4P was unable to bind to the modified pocket to induce conformational changes of the helix switch and as a consequence, the ATPase activity remained inhibited. Taken together, our structural and functional analyses strongly suggest that the positively charged pocket above the helix switch is the PI4P binding site in Drs2p. Indeed, a latest cryo-EM analysis of Drs2p-Cdc50p shows that either PI4P or PS may bind in this pocket^24^.

## Discussion

**Fig. 6:**
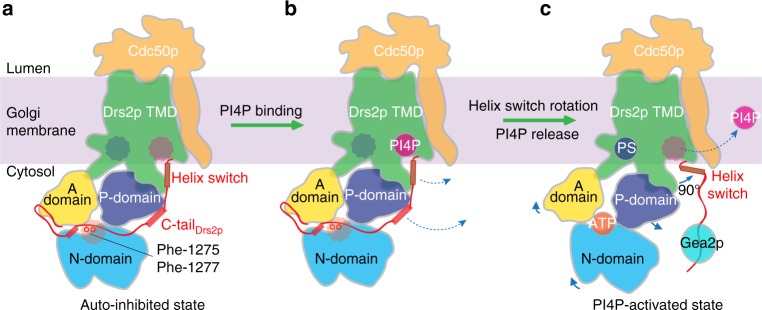
A model for Drs2p-Cdc50p autoinhibition and activation by PI4P. **a** In the absence of an activation factor, the Drs2p-Cdc50p flippase exists in the membrane in an autoinhibited state. Autoinhibition is primarily achieved by the extended C-terminus wrapping around the cytosolic A, P, and N domains. Specifically, Phe-1275 and Phe-1277 occupy the ATP-binding pocket, preventing access of ATP to the catalytic site. **b** Activation of the flippase is initiated by the binding of PI4P in a positively charged pocket in the transmembrane region proximal to the cytosol. **c** The binding of PI4P leads to a 90° rotation of the helix switch away from the cytosolic domains, thereby pulling the C-terminal inhibition peptide out of the cytosolic domains, and activating the enzyme. Upon Drs2p activation, ATP and the substrate PS bind to Drs2p, but PI4P diffuses away from the flippase. The active state is likely sustained by additional factors such as Gea2p, which is known to bind to the Drs2p C-terminal inhibition loop. The three circles mark the ATP, PS, and PI4P binding sites in Drs2p

Based on our cryo-EM structures reported here and other published mutational and functional studies^4,5,9,11,13^, we propose that self-inhibition of the Drs2p-Cdc50p complex is achieved primarily by the action of the long C-terminal peptide, which is connected to the last transmembrane helix TM10 via a helix switch in Drs2p. The C-terminal peptide inserts into a deep horizontal groove in the regulatory A, N, and P domains at the cytosolic side (Fig. 6a). In so doing, the C-terminal peptide holds these regulatory domains together, preventing them from the relative motions that are required for their function. In addition, two large hydrophobic residues in the C-terminal peptide, Phe-1275 and Phe-1277, insert into the ATP-binding pocket in the Drs2p N domain, thereby preventing ATP from entering its binding site.

We further propose that PI4P binds to a highly positively charged pocket in the cytosol-proximal region of the transmembrane domain in Drs2p (Fig. 6b). PI4P binding then triggers a 90° swing of the helix switch toward the right, which drags the inhibitory C-terminal peptide out of its binding groove and releases it, thereby activating the Drs2p ATPase (Fig. 6c). Dislodging the C-terminal peptide allows the A, N, and P domains to move and vacates the ATP-binding site, so the enzyme is ready for ATP binding and hydrolysis that drives substrate transport across the membrane. Our mechanisms of Drs2p-Cdc50p autoinhibition and the PI4P-mediated activation are highly similar to the most recent report^24^. However, PI4P remains bound in the C-terminal truncation-activated Drs2p-Cdc50p structure^24^, whereas in the full-length Drs2p-Cdc50, PI4P binds only transiently in the activation pocket and diffuses away from Drs2p once the enzyme is activated. How PI4P leaves the pocket is unknown, but we speculate that the 90° swing of the helix switch may do more than just pulling the C-terminal peptide out of its binding groove. The switch motion may distort the PI4P binding pocket enough to expel PI4P. If this is true, what would prevent the helix switch from reverting back and allowing the C-terminal peptide to re-insert into the groove? That is, what prevents the activated Drs2p from re-inhibiting itself? We suggest that there may be a high energy barrier to such a reversion, or perhaps more likely, that the freed C-terminal peptide may be stabilized in solution by an additional protein factor, such as Gea2p (Fig. 6c). Gea2p is known to bind a C-terminal region of Drs2p (Phe-1256 to Met-1269) and to contribute to the activation of the enzyme complex^9,10^. This region is inaccessible in the autoinhibited form when the C-terminus is bound to the cytosolic region of Drsp2 but should be fully exposed in the activated form for a stabilizing interaction with Gea2p.

In summary, our cryo-EM structures of the intact Drs2p-Cdc50p in the apo- and the PI4P-activated forms have elucidated the molecular mechanisms for the enzyme autoinhibition and the PI4P-mediated activation process. Further work is needed to understand how the activated conformation is sustained or deactivated, and how the complex flips phosphatidylserine from the Golgi lumen to the cytoplasmic side of the membrane.

## Methods

### Purification of the endogenous Drs2p-Cdc50p complex

The C-terminal triple FLAG-tagged Drs2 construct was generated using a PCR-based genomic epitope-tagging method on the yeast W303-1a (*MATa leu2-3*,*112 trp1-1 can1-100 ura3-1 ade2-1 his3-11*, obtained from the Michael O’Donnell Lab at Rockefeller University) (Supplementary Table 1). About 18 L of cells was cultured and then harvested in lysis buffer containing 20 mM Tris-HCl (pH 7.4), 0.2 M sorbitol, 50 mM potassium acetate, 2 mM EDTA, and 1 mM phenylmethylsulfonyl fluoride (PMSF). Cells were lysed using a French press at 15,000 psi and then were centrifuged at 10,000 × *g* for 30 min at 4 °C. The supernatant was then centrifuged at 100,000 × *g* for 60 min at 4 °C to collect the membrane pellet. The membrane was solubilized in buffer A containing 10% glycerol, 20 mM Tris-HCl (pH 7.4), 1.5% *n*-dodecyl β-D-maltoside (DDM), 0.15% cholesteryl hemisuccinate Tris salt (CHS), 0.5 M NaCl, 1 mM MgCl_2_, 1 mM MnCl_2_, 1 mM EDTA, and 1 mM PMSF. After incubation for 30 min at 4 °C, the mixture was centrifuged for 30 min at 120,000 × *g*, and the clarified supernatant was loaded into a home-packed anti-FLAG (M2) affinity gel column at 4 °C. The column was then washed three times in buffer B containing 0.025% DDM, 0.0025% CHS, 150 mM NaCl, 20 mM Tris-HCl, pH 7.4, 1 mM MgCl_2_, and 1 mM MnCl_2_. Finally, the Drs2p-Cdc50p complex was eluted with three column volumes of buffer B containing 0.15 mg/mL 3×FLAG peptide, concentrated to 500 μL with a 100-kDa cutoff centricon. The sample was further purified in a Superose 6 10/300 gel filtration column in buffer C containing 0.01% lauryl maltose-neopentyl glycol (LMNG), 0.001% CHS, 150 mM NaCl, 20 mM Tris-HCl, pH 7.4, 1 mM MgCl_2_, and 1 mM MnCl_2_. For the active Drs2p-Cdc50p, the purified sample in Buffer C was incubated with 26 μM PI4P for 15 h at 4 °C. The final sample was assessed by SDS-PAGE gel and the identities of the protein bands were confirmed by tryptic digestion followed by mass spectrometry.

### Cryo-electron microscopy

Aliquots of 3 μL of purified Drs2p-Cdc50p at a concentration of 3 mg/mL were placed on glow-discharged holey carbon grids (Quantifoil Au R2/1, 300 mesh) and were flash-frozen in liquid ethane using an FEI Vitrobot Mark IV. The micrographs were screened in a 200 kV FEI Arctica electron microscope. A small data set of 200 micrographs collected in this microscope on an FEI Falcon III direct detector led to some 120,000 raw particles. After 2D classification, about 70,000 particles were selected for 3D reconstruction, which resulted in a 6.2-Å preliminary 3D map. A full data set was collected during a 4-day session in a 300 kV FEI Titan Krios electron microscope operated at a nominal magnification of ×130,000 and a pixel size of 1.029 Å per pixel with defocus values from −1.0 to −2.0 μm. The dose rate was 2 electrons per Å^2^ per second per frame, and 40 frames were recorded in a movie.

### Image processing

The movie micrographs collected from the Titan Krios were motion-corrected using the program MotionCorr 2.0^25^. Contrast transfer function parameters of each aligned micrograph were calculated using CTFFIND 4.1^26^. All the remaining steps were performed using RELION 3^27^. For apo form, 2970 raw movie micrographs were collected. Templates for automatic picking were generated from a 2D average of about 1000 manually picked particles. A total of 1,040,625 particles were picked automatically. 2D classification was then performed and particles in the classes with features unrecognizable by visual inspection were removed. A total of 1,001,980 particles was used for further 3D classification, and 635,300 particles were selected for further 3D refinement and postprocessing, resulting in the 2.8-Å 3D density map. The resolution of the map was estimated by the gold-standard Fourier shell correlation at a correlation cutoff value of 0.143. For active form, 4717 raw movie micrographs were collected. A total of 1,126,540 particles were picked automatically. After 2D classification, a total of 897,023 particles was used for 3D classification, and 498,745 particles were selected for further 3D refinement and postprocessing, resulting in the 3.3-Å 3D density map.

### Structural modeling and refinement and validation

We first built the apo Drs2p-Cdc50p model into the 2.8-Å 3D density map. The initial model of Drs2p was generated based on PDB ID 5YLV using online SWISSMODEL (https://swissmodel.expasy.org). The model was split into four domains: TM, A, N, and P domains, which were then fitted into the EM density independently in Chimera^28^. Initial model of Cdc50p was first automatically built using the model_to_map in the PHENIX program^29^ and then was manually built in the program COOT^30^. The complete Drs2p-Cdc50p model was refined by real-space refinement in the PHENIX program and subsequently adjusted manually in COOT. Active Drs2p-Cdc50p model was then built into the 3.3-Å 3D density map basing on the apo model. Notably, the A and N domains of Drs2p were only built by rigid-body fitting because of their low local resolution at 4–6 Å. Finally, the atomic model was validated using MolProbity^31^. To avoid overfitting, the 3D maps of the final map and the two half-maps (Half1 and Half2) were correlated with the refined model to produce three FSC curves: Model vs. final map, FSC_work_ (Model vs. Half1 map), and FSC_free_ (Model vs. Half2 map). Structural figures were prepared in Chimera^28^ and PyMOL (https://pymol.org/2/).

### ATPase assay

Purified Drs2p-Cdc50p was first incubated with 26 μM PI4P for 15 h at 4 °C, and then assayed for ATPase hydrolysis activity in buffer containing 20 mM HEPES, pH 7.5, 150 mM NaCl, 0.01% LMNG, 0.001% CHS, 10 mM MgCl_2_ at 37 °C for 1 h. The sample without PI4P incubation was used as control. Released phosphate was measured colorimetrically using Malachite Green Phosphate Assay Kit from Sigma.

### Complementation assay of *drs2*Δ cells

Yeast *drs2*Δ strain and the complement plasmid carrying *drs2* gene were gifts from Prof. Todd Graham in Vanderbilt University. Drs2 mutants were generated by QuikChange mutagenesis protocol with Pfu DNA polymerase and desired mutation primers (Supplementary Table 1). To determine the growth difference between *drs2*Δ yeast transformants carrying either wide-type *drs2* plasmid or mutations, the strains firstly grew to the same OD in + G418 YPD medium at 30 °C. Then 7 μL of 1:10 serial dilutions of the cells were spotted onto + G418 YPD plates, incubated at 20 or 30 °C for 2 days, and examined for growth.

### Reporting summary

Further information on research design is available in the Nature Research Reporting Summary linked to this article.

## Supplementary information

Supplementary Information

Description of Additional Supplementary Files

Supplementary Movie 1

Reporting Summary

Source Data

## Data Availability

Data supporting the findings of this manuscript are available from the corresponding authors upon reasonable request. A reporting summary for this Article is available as a Supplementary Information file. The source data underlying Supplementary Fig. 1 is provided as a Source Data file. The structures of the Drs2p-Cdc50p complex in the autoinhibited apo form and in the PI4P-activated form have been deposited in the Electron Microscopy Data Bank and Protein Data Bank, under accession codes EMD-20468 and PDB ID 6PSY (apo form), and EMD-20467 and PDB ID 6PSX (active form), respectively.
