# Supplementary material for: Autoinhibition and activation mechanisms of the eukaryotic lipid flippase Drs2p-Cdc50p
Source: Nat Commun. 2019 Sep 12;10:4142. doi: 10.1038/s41467-019-12191-9 (PMC6742660; doi:10.1038/s41467-019-12191-9)
Supplement: Supplementary file 1 — Supplementary Information [file 41467_2019_12191_MOESM1_ESM.pdf]

Supplementary information

Autoinhibition and activation mechanisms of the eukaryotic lipid flippase Drs2p-Cdc50p

Bai et al.

Supplementary Table 1. Primer sequences for generating *Drs2* FLAG tagging and point mutations

	**Forward primer** (5'-> 3')	**Reverse primer** (5'->3')
FLAG-tag	AAAATTCAAATAGATTCAGTTCTTCGAGAG ATGATATTTCATTTGATATACGGATCCCCG GGTTAATTAA	TCTTATTTTTATTTTTGTATTTTTTGTC TTCTGCTCTTTGTTTCTGTGCCTGAATT CGAGCTCGTTTAAAC
W1223A	CACTGGTAAGAGATTTTCTAGCGAAGTACT ATAAAAGAATG	CATTCTTTTATAGTACTTCGCTAGAAAA TCTCTTACCAGTG
K1227A	GATTTTCTATGGAAGTACTATGCAAGAAT GTATGAACCAGAAACG	CGTTTCTGGTTCATACATTCTTGCATA GTACTTCCATAGAAAATC
R1228A	GATTTTCTATGGAAGTACTATAAAGCAAT GTATGAACCAGAAACG	CGTTTCTGGTTCATACATTGCTTTATAG TACTTCCATAGAAAATC
Y1235A	GAATGTATGAACCAGAAACGGCTCATGTT ATTCAAGAAATGCAG	CTGCATTTCTTGAATAACATGAGCCGTT TCTGGTTCATACATTC
H1236A	GAATGTATGAACCAGAAACGTATGCTGTTA TTCAAGAAATGCAG	CTGCATTTCTTGAATAACAGCATACGT TTCTGGTTCATACATTC

Supplementary Table 2. Cryo-EM data collection, refinement, and validation statistics for the *S. cerevisiae* Drs2p-Cdc50p complex

	Drs2p-Cdc50p Auto-inhibited apo (EMD-20468, PDB 6PSY)	Drs2p-Cdc50p PI4P-activated (EMD-20467, PDB 6PSX)
**Data collection and processing**		
Microscope	FEI Titan Krios	FEI Titan Krios
Voltage (kV)	300	300
Electron exposure ($e^{-}/Å^{2}$)	80	80
Defocus range (μm)	-1.0– -2.0	-1.0 – -2.0
Pixel size (Å)	1.029	1.029
Symmetry imposed	C1	C1
Initial particle images (no.)	1040625	1126540
Final particle images (no.)	635300	498745
Map resolution (Å)	2.8	3.3
FSC threshold	0.143	0.143
Map resolution range (Å)	247.0-2.8	247.0-3.3
**Refinement**		
Map sharpening *B* factor ($Å^{2}$)	108	148
Model composition		
Non-hydrogen atoms	11457	11065
Protein residues	1412	1358
N-glycans	3	3
R.m.s. deviations		
Bond lengths (Å)	0.01	0.01
Bond angles (°)	1.11	1.10
Validation		
MolProbity score	1.99	1.95
Clashscore	9.02	8.00
Poor rotamers (%)	0.24	0.33
Ramachandran plot		
Favored (%)	90.7	91.0
Allowed (%)	9.3	9.0
Disallowed (%)	0	0

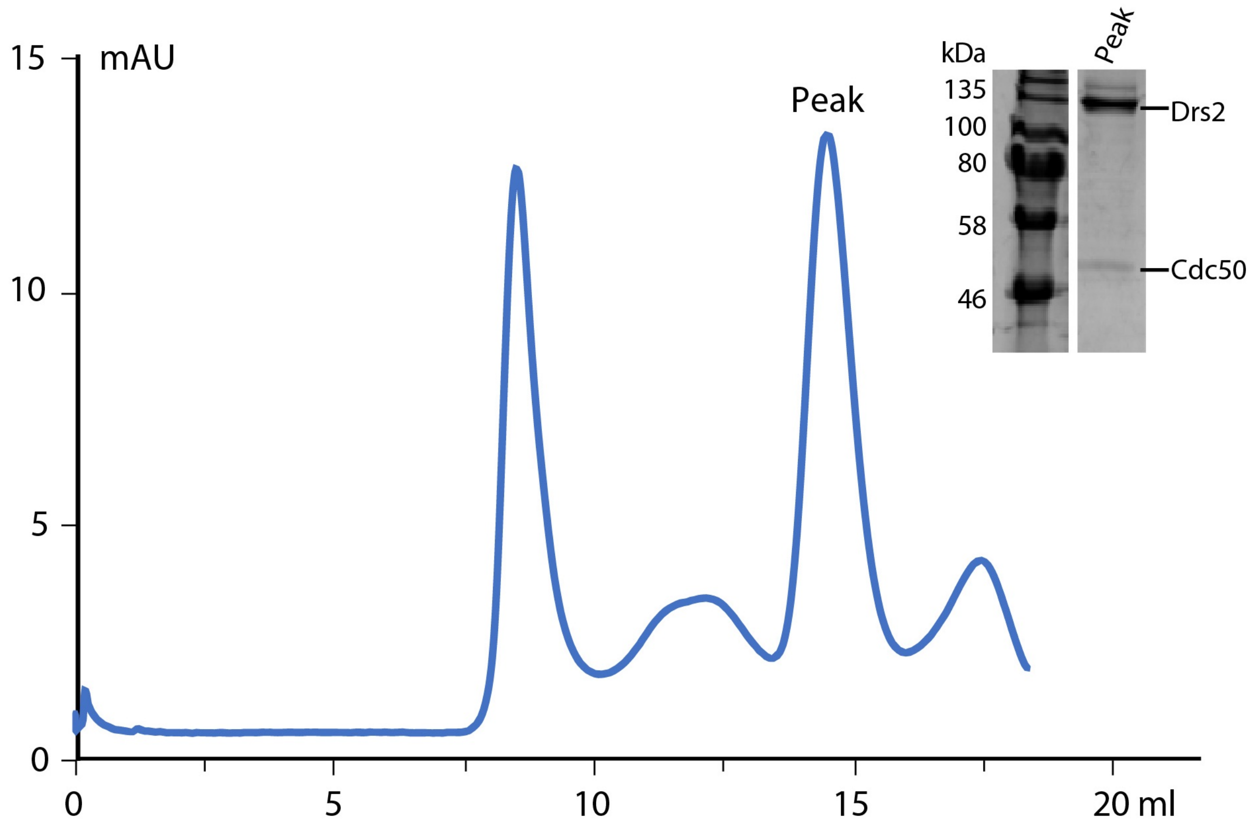

Supplementary Figure 1. Purification of the yeast Drs2p-Cdc50p complex. Gel filtration and the Coomassie blue-stained SDS-PAGE gel of the Drs2p-Cdc50p complex. Source data for the SDS-PAGE insert is provided as a Source Data file.

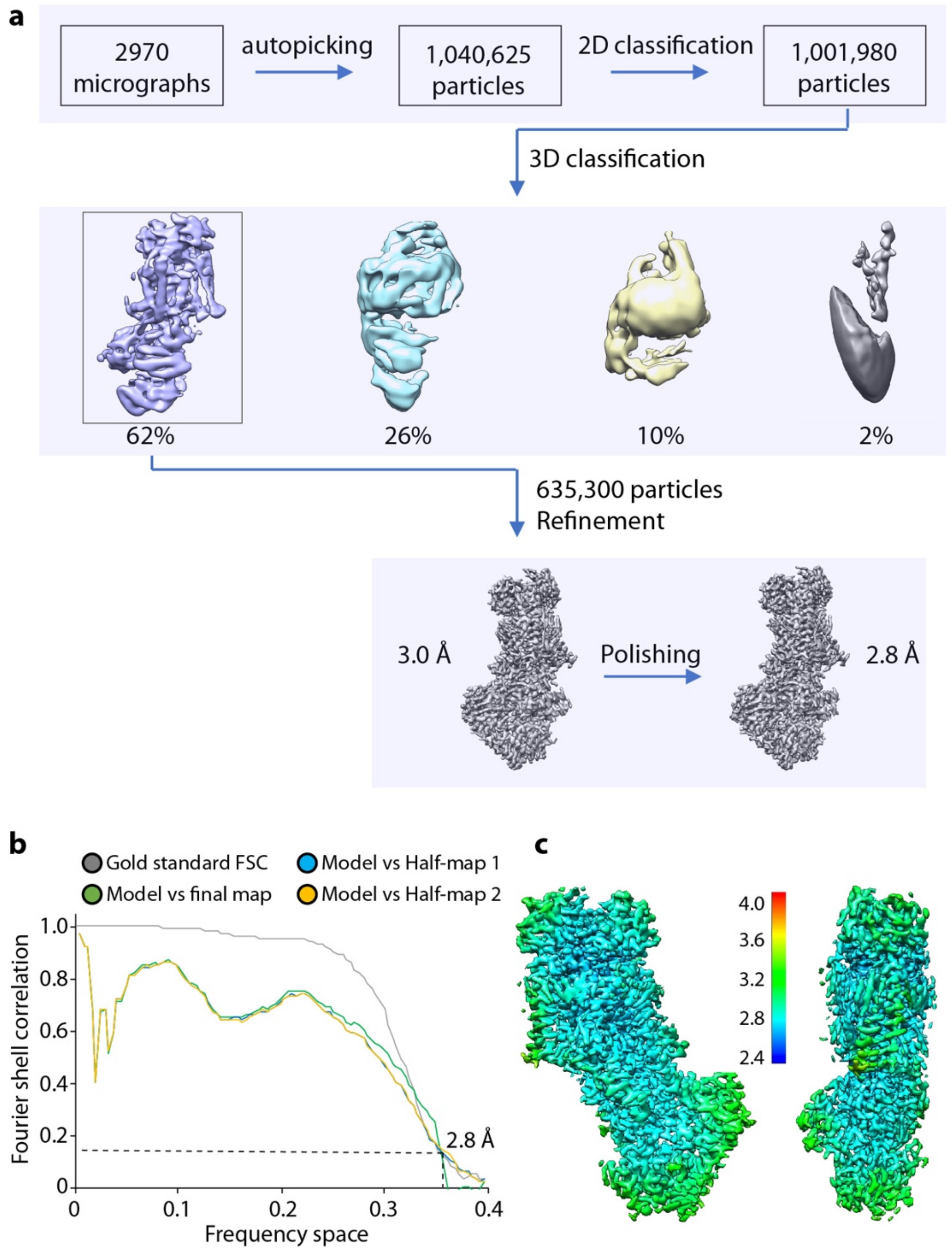

Supplementary Figure 2. Cryo-EM data processing and validation of the apo Drs2p-Cdc50p complex. (**a**) Cryo-EM data processing procedure. (**b**) Gold-standard Fourier correlation of two independent half maps, and the validation correlation curves of the atomic model by comparing the model with the final map or with the two half maps. (**c**) Local resolution map of the Drs2p-Cdc50p complex structure in two side views.

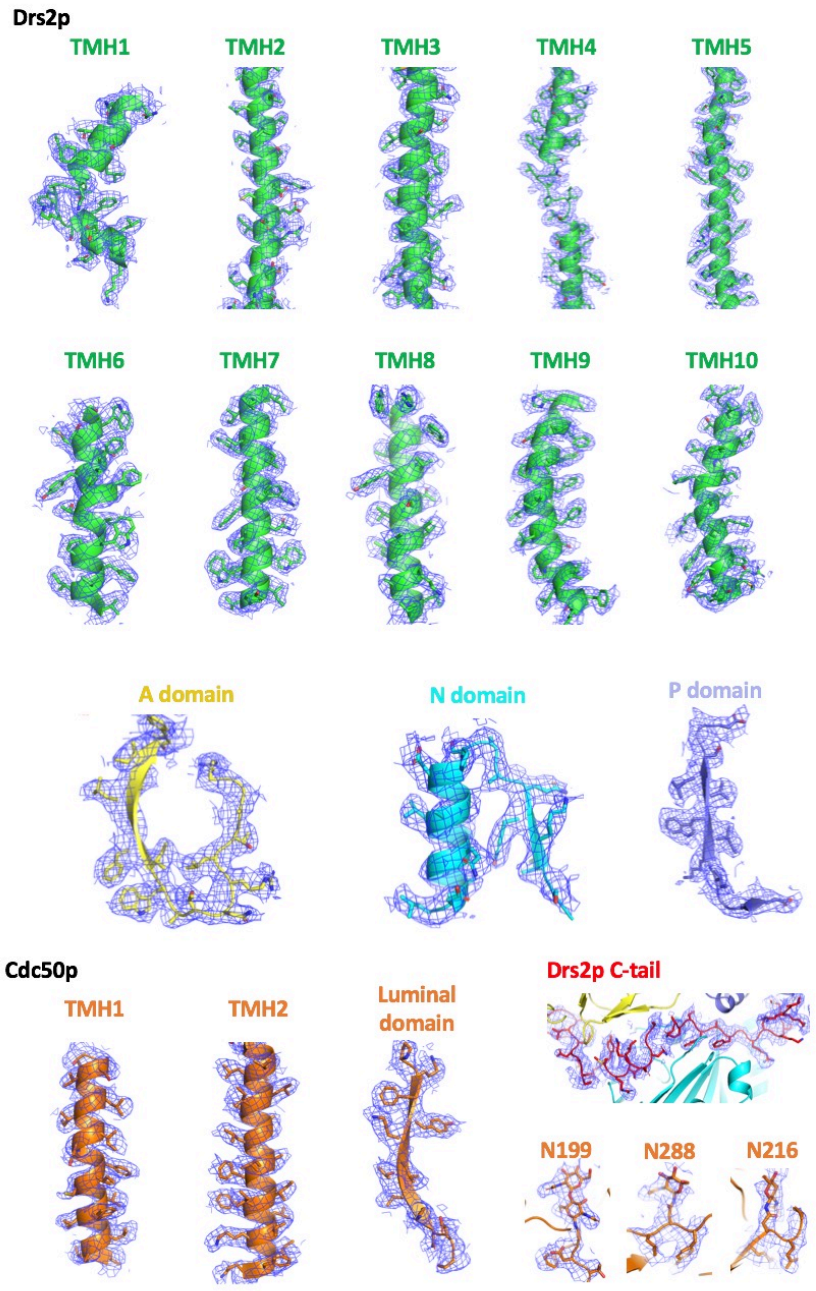

Supplementary Figure 3. 3D density map and atomic model of selected regions in the apo Drs2p-Cdc50p structure. The display threshold is about 3σ.

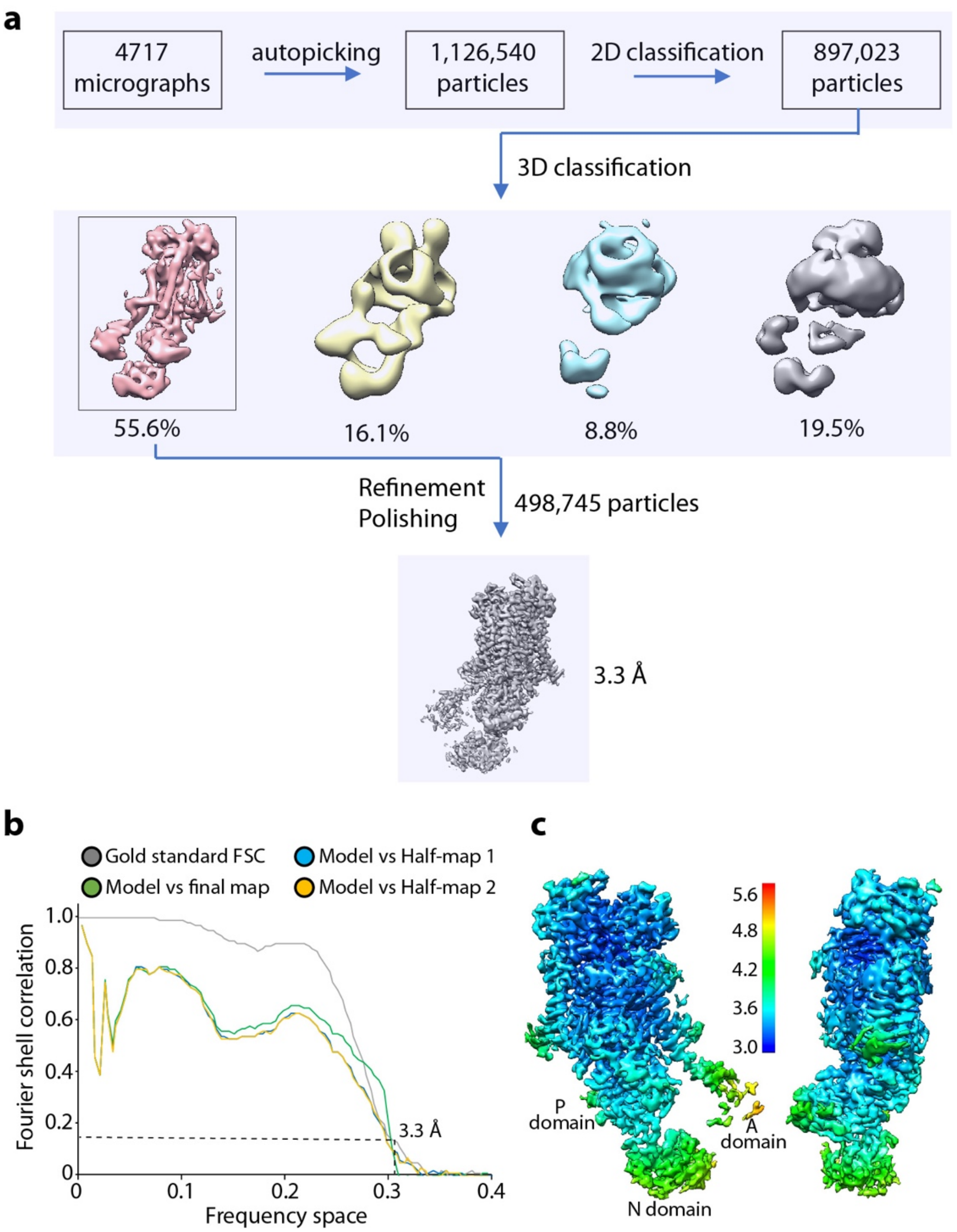

Supplementary Figure 4. Cryo-EM data processing and validation of the PI4P-activated Drs2p-Cdc50p complex. (**a**) Cryo-EM data processing procedure. (**b**) Gold-standard Fourier correlation of two independent half maps, and the validation correlation curves of the atomic model by comparing the model with the final map or with the two half maps. (**c**) Local resolution map of the activated structure. The A domain becomes flexible in the activated conformation.

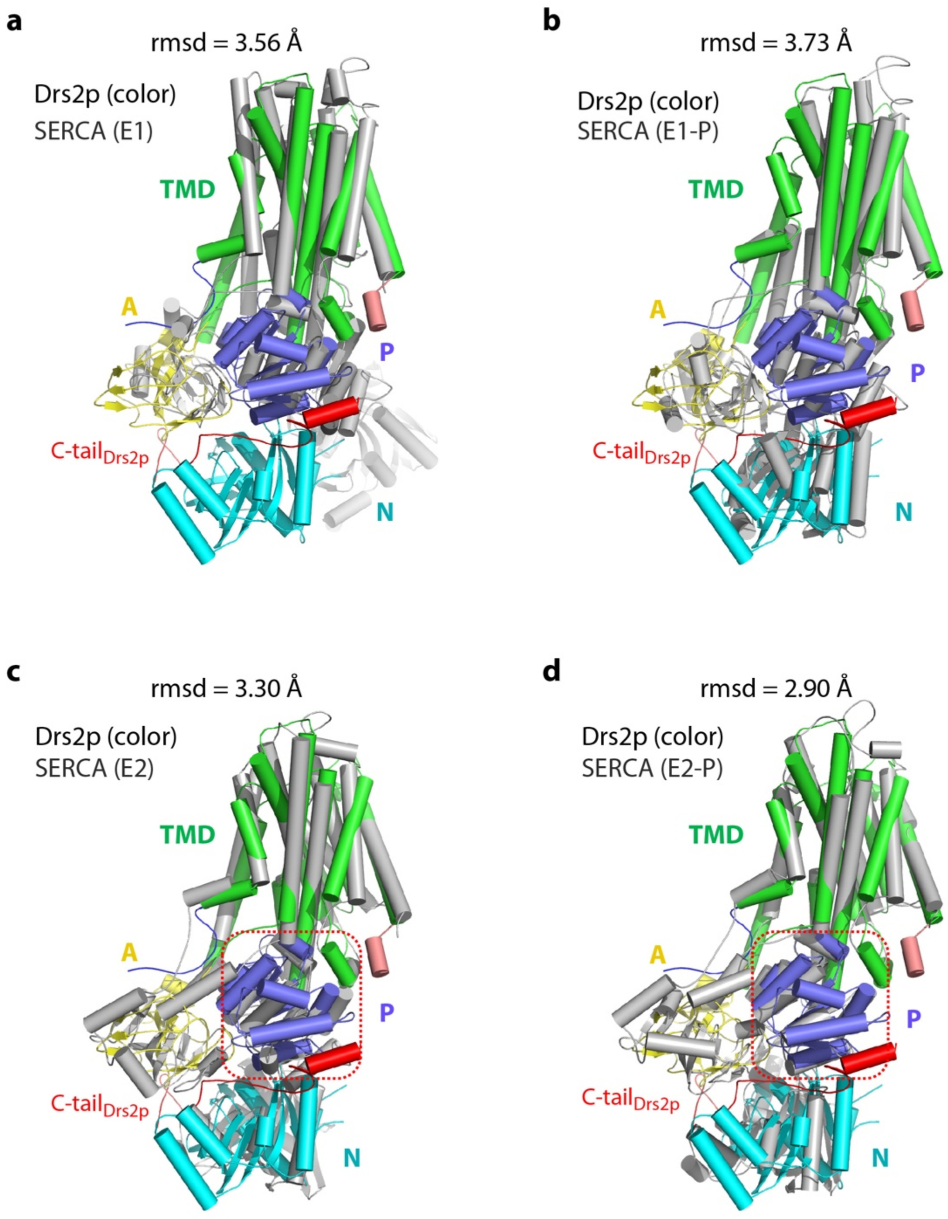

Supplementary Figure 5. Structural comparison of Drs2p in apo form with the sarco(endo)plasmic reticulum Ca^{2+}-ATPase (SERCA) in four functional states. The SERCA ATPase in the E1 state (PDB 5XA7) (**a**), E1-P state (PDB 5XA8) (**b**), E2 state (PDB 5XAB) (**c**), and E2-P state (PDB 5XAA) (**d**) are aligned with Drs2p by their respective TMDs. Red boxes in (c, d) highlight the P domains that superimpose well between Drs2p and the SERCA ATPase in the E2 and E2-P states. The rmsd value is estimated between the aligned transmembrane regions in each state. The positions of the A, N, and P domains are very different and their alignment rmsd values are not shown.

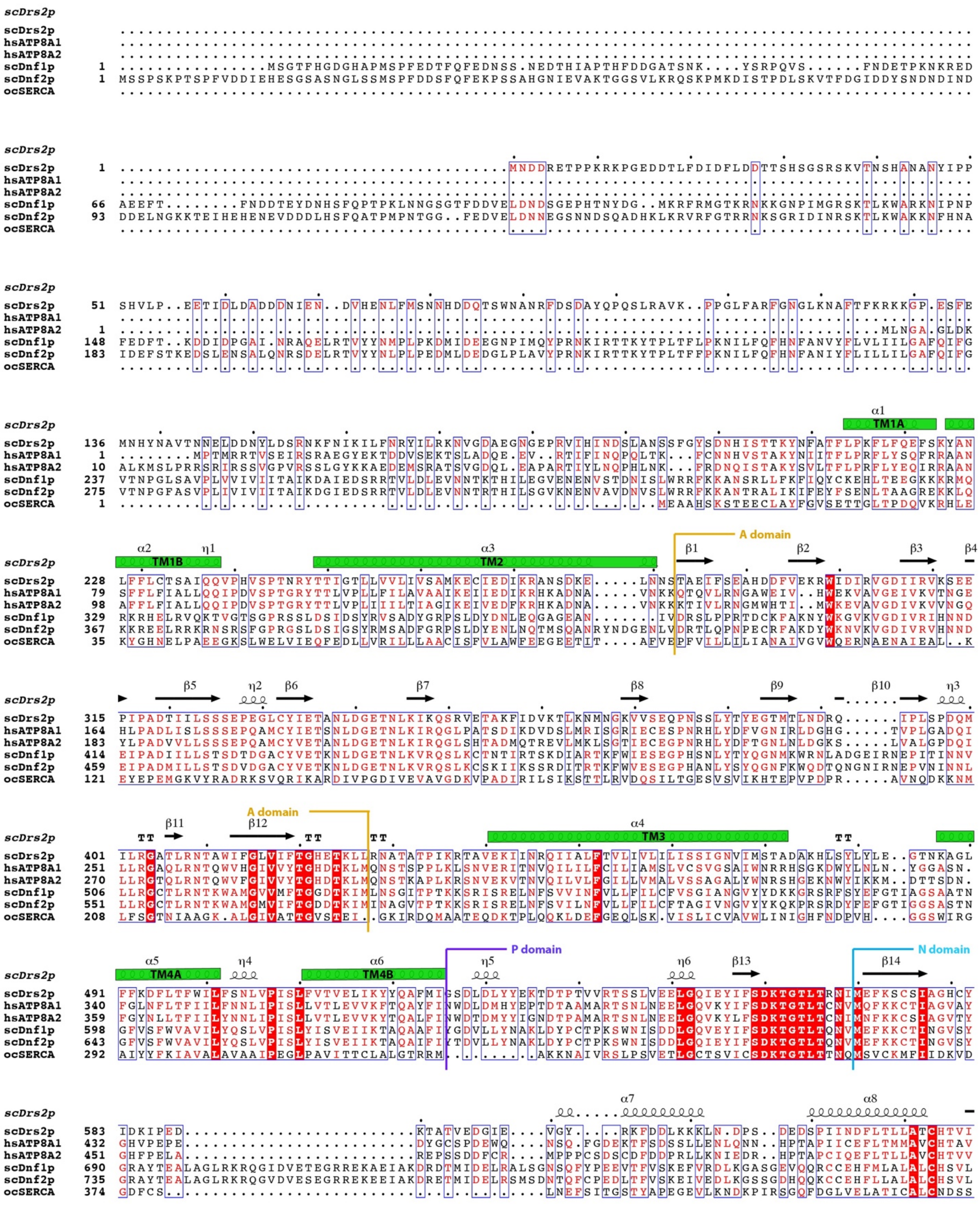
scDrs2p
hsATP8A1
hsATP8A2
scDnf1p
scDnf2p
ocSERCA
TM1A
TM1B
TM2
TM3
TM4A
TM4B
A domain
P domain
N domain

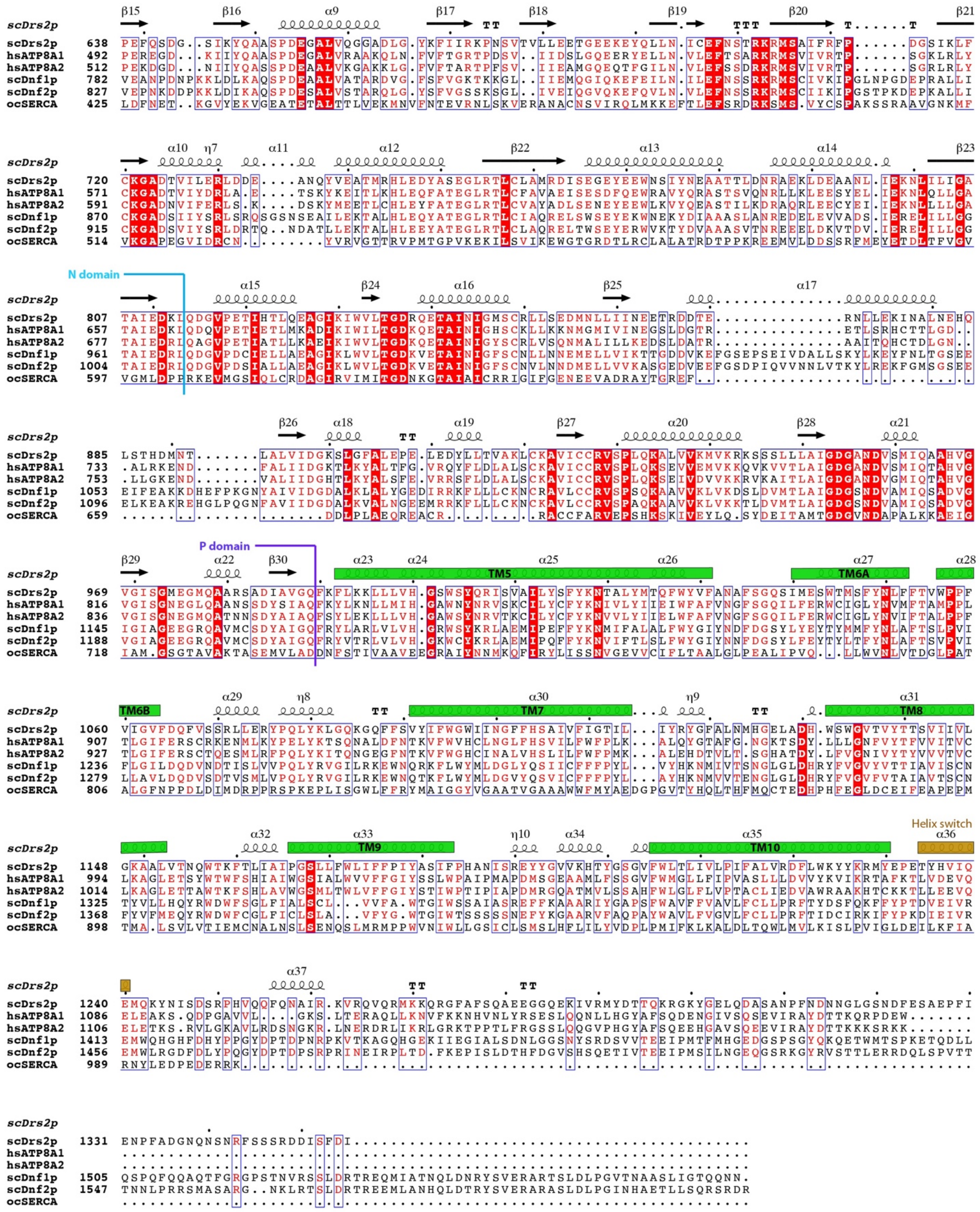

Supplementary Figure 6. Sequence alignment of selected eukaryotic Drs2p. hs, *Homo sapiens*; sc, *Saccharomyces cerevisiae*; oc, *Oryctolagus cuniculus*. The positions of the 10 transmembrane helices, the domain boundaries for domains A, N, and P, and the helix switch of Drs2p are labeled above the sequence.

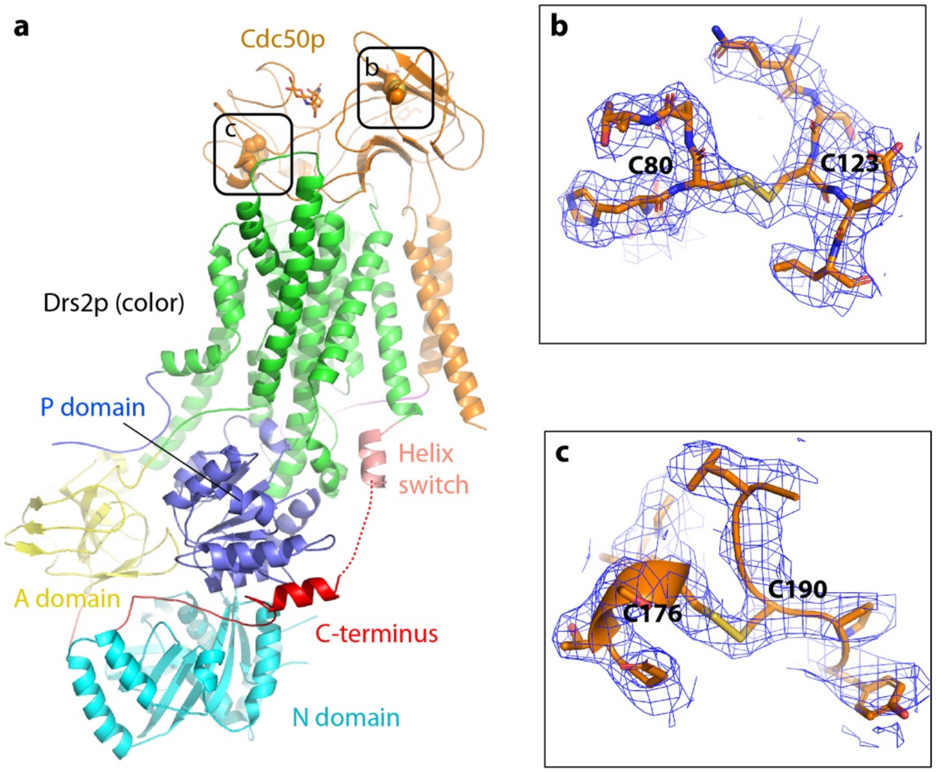

Supplementary Figure 7. Two disulfide bonds (Cys-80 with Cys-123 and Cys-176 with Cys-190) in the lumenal domain of Cdc50p. (a) Overall structure of the Drs2p-Cdc50p in a side view. The four cysteine residues forming two disulfide bonds are shown as brown spheres and the two regions containing the disulfide bonds are marked by two black squares. **(b, c)** Enlarged views of the boxed regions in (a), showing the disulfide bonds in sticks between Cys-80 and Cys-123 (b) and between Cys-176 and Cys-190 (c), superimposed with the cryo-EM densities in blue mesh displayed at a threshold of 3σ.

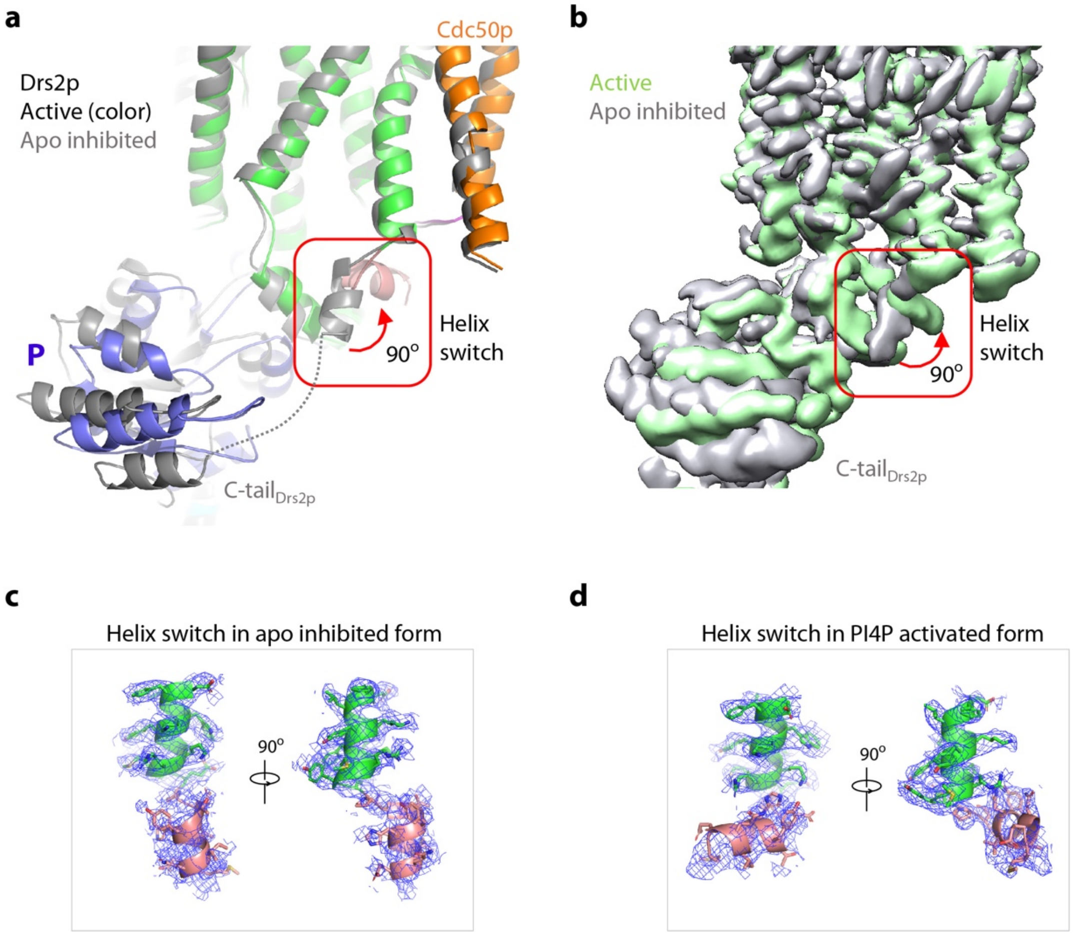

Supplementary Figure 8. A 90° rotation of the helix switch upon PI4P binding releases the C-terminus and activates Drs2p. (**a**) Superimposition of the autoinhibited apo state (gray) and the PI4P-activated state (color) of Drs2p in cartoon presentation. (**b**) Superimposed 3-D maps of the autoinhibited apo state (gray) and the PI4P-activated state (green) of Drs2p. The 3D densities shown here are refined but before post-processing. Post-processing makes the helix switch density somewhat weaker. In (a, b), the red box highlights the helix switch region, and the curved red arrow indicates the 90° rotation of the helix switch. (**c, d**) 3D density for the helix switch displayed in blue mesh at a threshold of 3.0σ in the apo form (c) and 4.5σ in the activated form (d).
