# Supplementary material for: Autoinhibition and activation mechanisms of the eukaryotic lipid flippase Drs2p-Cdc50p
Source: Nat Commun. 2019 Sep 12;10:4142. doi: 10.1038/s41467-019-12191-9 (PMC6742660; doi:10.1038/s41467-019-12191-9)
Supplement: Supplementary file 2 — Description of Additional Supplementary Files [file 41467_2019_12191_MOESM2_ESM.pdf]

Description of Additional Supplementary Files

File name: Supplementary Movie 1

Description: A 360° rotation around a vertical axis of the surface-rendered cryo-EM 3D map of the yeast Drs2p-Cdc50p complex. Subunits and major domains of Drs2p are labeled.
