# Supplementary material for: Autoinhibition and activation mechanisms of the eukaryotic lipid flippase Drs2p-Cdc50p
Source: Nat Commun. 2019 Sep 12;10:4142. doi: 10.1038/s41467-019-12191-9 (PMC6742660; doi:10.1038/s41467-019-12191-9)
Supplement: Supplementary file 5 — Source Data [file 41467_2019_12191_MOESM5_ESM.docx]

Source Data file for

**The autoinhibition and the activation mechanisms of the eukaryotic lipid flippase Drs2p-Cdc50p**

Lin Bai, Amanda Kovach, Qinglong You, Hao-Chi Hsu, Gongpu Zhao, and Huilin Li

This file contains the original SDS-PAGE used in Supplementary Figure 1


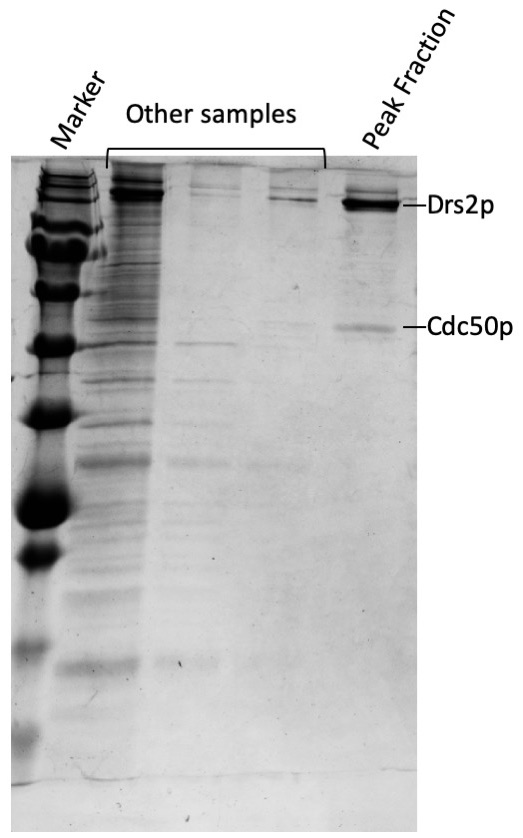


Source data for the SDS-PAGE gel used in Supplementary Figure 1.
